# Dr. H. F. Nichols

**Published:** 1915-03

**Authors:** 


**Dr. H. F. Nichols,** (not listed in State Directory or Polk) died at his home in Wethersfield Springs, N. Y., Feb. 6, aged 68. He had practiced in Attica for over 30 years, until his retirement 2 years ago.

